# Supplementary material for: ROMP and Vinyl Polynorbornenes with Vanadium(III) and Nickel(II) diNHC Complexes
Source: Int J Mol Sci. 2025 Jul 12;26(14):6691. doi: 10.3390/ijms26146691 (PMC12294648; doi:10.3390/ijms26146691)

Supplementary Materials

ROMP and Vinyl Polynorbornenes with Vanadium(III) and Nickel(II) diNHC Complexes

Katarzyna Halikowska-Tarasek [1], Elwira Bisz [1], Dawid Siodłak[1], Błażej Dziuk [2] and Wioletta Ochędzan-Siodłak [1,*]

[1] Department of Chemistry and Pharmacy, Opole University, Oleska 48, Opole 45-052, Poland

[2] Department of Chemistry, Wroclaw University of Science and Technology, Norwida 4/6, Wroclaw 50-373, Poland

Correspondence: wsiodlak@uni.opole.pl

Table of Contents

Experimental Section 4

Synthesis of Imidazolium Salts 4

Synthesis of Vanadium(III) Complexes 5

Synthesis of Nickel(II) Complexes 5

Figure S1. Photographic representation of the starting solution and the final products 6

Norbornene Polymerization Catalyzed by Vanadium(III) Complexes 7

Figure S2. Photographic representation of the final polynorbornene products obtained using vanadium(III) catalysts 7

Norbornene Polymerization Catalyzed by Nickel(II) Complexes 8

Figure S3. Photographic representation of the final polynorbornene products obtained using nickel(II) catalysts 8

References 9

^{1}H and ^{13}C NMR Spectra 10

Figure S4. ^{1}H and ^{13}C NMR of bis(imidazolium) salt 2a 10

Figure S5. ^{1}H and ^{13}C NMR of polynorbornene obtained using vanadium(III) catalyst 3a 11

Figure S6. ^{1}H and ^{13}C NMR of polynorbornene obtained using vanadium(III) catalyst 3b 12

Figure S7. ^{1}H and ^{13}C NMR of polynorbornene obtained using nickel(II) catalyst 4a 13

Figure S8. ^{1}H and ^{13}C NMR of polynorbornene obtained using nickel(II) catalyst 4b 14

MS spectra 15

Figure S9. MS spectrum of pre-ligand 2a 15

Figure S10. MS spectrum of vanadium(III) catalyst 3a 15

Figure S11. MS spectrum of vanadium(III) catalyst 3b 15

Figure S12. MS spectrum of nickel(II) catalyst 4a 16

Figure S13. MS spectrum of nickel(II) catalyst 4b 16

FTIR spectra 17

Figure S14. FTIR (ATR) of polynorbornene obtained using vanadium(III) catalyst 3b 17

Figure S15. FTIR (ATR) of polynorbornene obtained using nickel(II) catalyst 4b 17

DSC and TGA analysis 18

Figure S16. Tm of PNB obtained using catalyst 3b 18

Figure S17. Tg of PNB obtained using catalyst 4a 18

Figure S18. Tg of PNB obtained using catalyst 4b 18

Figure S19. TGA analysis of PNB obtained using catalyst 4a 19

Figure S20. TGA analysis of PNB obtained using catalyst 4b 19

GPC Analysis 20

Figure S21. GPC Report of PNB obtained using catalyst 3a 20
Figure S22. GPC Report of PNB obtained using catalyst 3b 21
Figure S23. GPC Report of PNB obtained using catalyst 4a 22
Figure S24. GPC Report of PNB obtained using catalyst 4b 23
SEM imaging 24
Figure S25. SEM images of PNB obtained using catalyst 4a 24
Figure S26. SEM images of PNB obtained using catalyst 4b 25

Experimental Section

All reactions were carried out under an inert (argon) atmosphere using Schlenk technique and glovebox. Toluene and THF were dried over sodium/benzophenone and distilled under nitrogen prior to use. Norbornene (99%) and dichloromethane were purchased from Thermo Scientific, MMAO-12, $AlEt_2Cl$ and $VCl_3(THF)_3$ were purchased from Sigma-Aldrich, and $NiCl_2(DME)$ was purchased from AmBeed, and were deoxygenated prior to use. Compounds 1 and 2b have been previously reported in the literature [34].

^1H NMR and ^{13}C NMR spectra were recorded on Bruker spectrometer at 400 (^1H NMR) and 100 MHz (^{13}C NMR). The spectra recorded in $CDCl_3$ (TMS internal standard) at room temperature. Fourier transform infrared spectroscopy (FTIR-ATR) was performed on a Thermo Nicolet NEXUS FTIR spectrometer in absorption mode using 10 scans. The spectra were collected in the 600-4000cm^{-1} range and a resolution was 2cm^{-1}. Mass spectroscopy (MS) was performed on a Waters Xevo G3 Q-TOF instrument. Thermal properties were determined by differential scanning calorimetry (DSC), analysis was conducted on a Mettler Toledo DSC 2010. Thermal stability was determined by thermogravimetric analysis (TG/TGA) using a TGA 2050 analyzer (TA Instruments). Molecular weight and molecular weight distribution of polymers were determined using a gel permeation chromatography system with a multiangle laser light scattering detector (GPC MALLS, DAWN HELEOS WYATT Technologies) and a refractive index detector (WGE Dr Bures, Dn-2010).

Synthesis of Imidazolium Salts

Imidazolium salts were synthesized according to our previously published procedure [34]. To a solution of imidazole **1** (0.56 mmol) in acetonitrile (0.56 mL) in a closed pressure tube, CH_2Br_2 or $C_4H_8Cl_2$ (0.28 mmol) was added. The mixture was heated at 110 °C for 3 days, leading to the formation of a precipitate. The precipitate was filtered and washed with diethyl ether until the supernatant was colorless. Then, the products (**2a**-**2b**) were dried in vacuo to give the desired bis(imidazolium) salt. All characteristics of salt **2b** are described in the Supporting Information of our article [34].

2a: New compound. The product was obtained in 53% yield (157.8 mg, 0.15 mmol) as a white solid of mp 284 – 285 ℃. ^1H NMR (400 MHz, $CDCl_3$) δ 10.68 (s, 2H), 7.42 (t, J = 7.6 Hz, 8H), 7.31 – 7.29 (m, 8H), 7.24 (d, J = 6.9 Hz, 8H), 7.10 (d, J = 7.4 Hz, 8H), 6.91 (d, J = 7.7 Hz, 8H), 6.79 (s, 4H), 5.04 (s, 4H), 2.41 (s, 6H), 2.23 (s, 6H), 1.67 (s, 2H), 1.07 (s, 6H). ^{13}C NMR (101 MHz, $CDCl_3$) δ 141.88, 141.77, 140.55, 140.46, 139.20, 130.66, 129.58, 129.43, 129.23, 128.87, 128.18, 127.81, 127.47, 52.30, 21.97, 10.22, 7.39. HRMS (ESI/Q-TOF) m/z (%) [M-2Br]$^{2+}$ calcd for $C_{77}H_{70}N4$ 525.2800 found 525.2782.

Synthesis of Vanadium(III) Complexes

Vanadium(III) complexes were synthesized according to modified published procedure [52-54]. Bis(imidazolium) salt **2a** or **2b** (0.99 mmol, 1 equiv.) was dissolved in THF (34 mL) and stirred. Then, 1.74 equiv. of solid $K[N(SiMe_3)_2]$ (343 mg, 1.72 mmol) was added. The resulting yellow solution was allowed to stir at RT for 48 h. After that time, it turned brown, and was filtered through Celite, using Schlenk technique. Then, the mixture was cooled to -20℃, and beige precipitate was formed, which was washed with cold hexane and dried under vacuum (Ar/vac). The beige intermediate products (carbenes) were obtained with high yield (70%). Then, carbene (0.69 mmnol, 1 equiv.) was dissolved in THF (10 mL), and the mixture was cooled to -78 °C, and 1 equiv. of $VCl_3(THF)_3$ (258 mg, 0.69 mmol) in THF (13 mL) was added to the resultant solution and stirred for 20 h. The resultant suspension was filtered through Celitea, **3a** was isolated as a beige solid in 50% yield after being dried under vacuum (Ar/vac), and **3b** was isolated as a brown solid in 71% yield after being dried under vacuum (Ar/vac).

3a: New compound. HRMS (ESI/Q-TOF) m/z (%) $[M-3Cl]^{3+}$ calcd for $C_{77}H_{70}VN_4$ 367.1680 found 367.1862.

3b: New compound. HRMS (ESI/Q-TOF) m/z (%) $[M-3Cl+3H]^{3+}$ calcd for $C_{80}H_{76}VN_4$ 382.1915 found 382.1798.

Synthesis of Nickel(II) Complexes

Nickel(II) complexes were synthesized according to modified published procedure [52,53,58,59]. Bis(imidazolium) salt **2a** or **2b** (0.99 mmol, 1 equiv.) was dissolved in THF (1 mL) and stirred. A mixture of 1.25 equiv. of KOtBu (139 mg, 1.24 mmol) suspended in THF (1mL), and using syringe was added to the flask. The light brown solution was stirred for 1 h and was filtered through Celite, using Schlenk technique. Then, the mixture was cooled to -78 ℃, and 1 equiv. of $NiCl_2(DME)$ (218 mg, 0.99 mmol) in THF (1mL) was added to the resultant solution and stirred for 1.5 h. The resultant suspension was filtered through Celite, **4a** was isolated as a gray solid in 50% yield after being dried under vacuum (Ar/vac), and **4b** was isolated as a green solid in 60% yield after being dried under vacuum (Ar/vac).

4a: New compound. HRMS (ESI/Q-TOF) m/z (%) $[M-2Cl+2H]^{2+}$ calcd for $C_{78}H_{74}NiN_4$ 563.2712 found 563.1896.

4b: New compound. HRMS (ESI/Q-TOF) m/z (%) $[M-2Cl]^{2+}$ calcd for $C_{80}H_{76}NiN_4$ 575.2712 found 575.3338.

Figure S1. Photographic representation of the starting solution and the final products

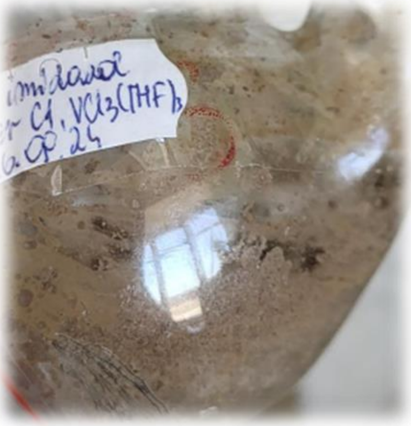

3a

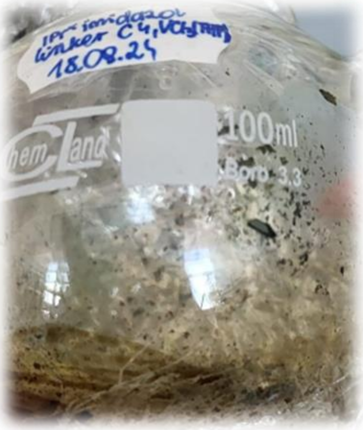

3b

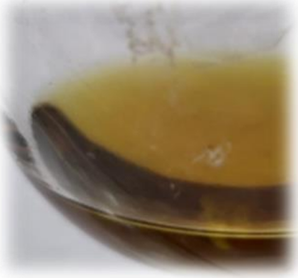

2a or **2b**

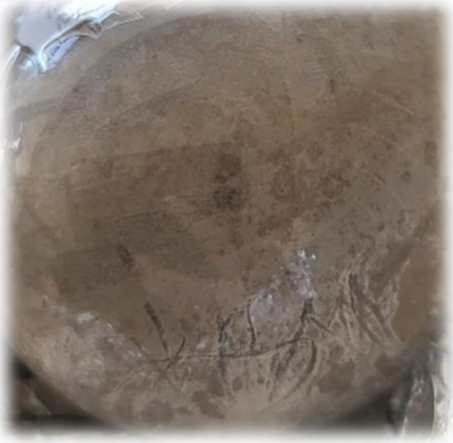

4a

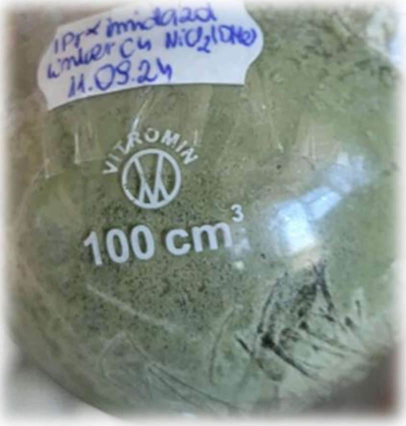

4b

Norbornene Polymerization Catalyzed by Vanadium(III) Complexes

In flask, norbornene (0.5 mol/dm^3) was dissolved in toluene (20 mL), then vanadium(III) catalyst (3×10^{-6} mmol) in CH_2Cl_2 (1 mL) was added. Subsequently, $AlEt_2Cl$ (diethylaluminium chloride, 1M in hexane, 1×10^{-3} mol/dm^3, 5 mL), and ETA (ethyl trichloroacetate, 97%, 2.2× 10^{-4} mol, 0.034 mL) were added into the flask by the syringes as an activator and reactivator, respectively. Then the flask was sealed and the mixture solution was stirred at RT for 48 h. Methanol was added to the polymerization mixture to terminate the reaction. The resultant polymer was obtained as yellow oil, later washed by hexane, and then by methanol, and dried. The reactions were repeated to ensure reproducibility of the results.

Figure S2. Photographic representation of the final polynorbornene products obtained using vanadium(III) catalysts

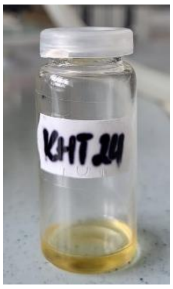

PNB obtained using **3a** catalyst

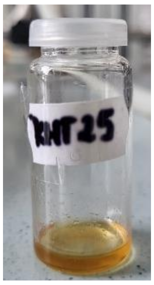

PNB obtained using **3b** catalyst

Norbornene Polymerization Catalyzed by Nickel(II) Complexes

In flask, norbornene (0.5 mol/dm^3) was dissolved in toluene (20 mL), then nickel(II) catalyst (3×10^{-6} mmol) in CH_2Cl_2 (1 mL) was added. Subsequently, MMAO-12 (modified methylaluminoxane, 7% in toluene, 3×10^{-3} mol/dm^3, 2.8 mL) was added by the syringe into the flask as a co-catalyst. The flask was sealed and the mixture solution was stirred at RT for 48 h. Methanol was added to the polymerization mixture to terminate the reaction. The resultant precipitated polymer was obtained by filtration, washed by hexane, and then by methanol, and dried to constant mass. The reactions were repeated to ensure reproducibility of the results.

Figure S3. Photographic representation of the final polynorbornene products obtained using nickel(II) catalysts

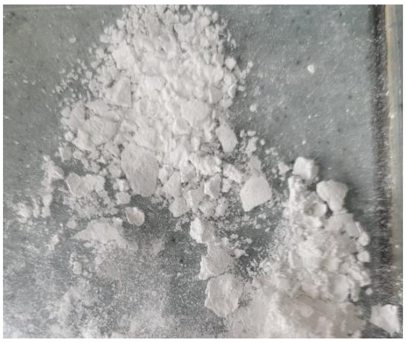

PNB obtained with catalyst 4a

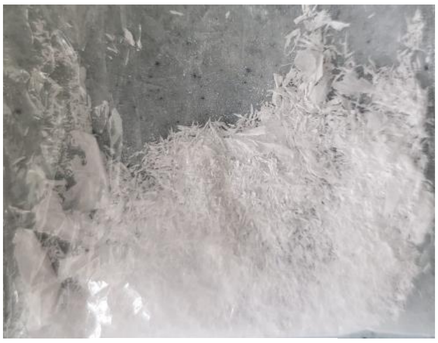

PNB obtained with catalyst 4b

References according to main text

[34] Halikowska-Tarasek, K.; Ochędzan-Siodłak, W.; Dziuk, B.; Szostak, R.; Szostak, M.; Bisz, E., IPr*^{diNHC}: Sterically Adaptable Dinuclear N-Heterocyclic Carbenes. *Inorg. Chem.* **2025**, 64, 16, 7851–7857.

[53] Kreisel, K. A.; Yap, G. P. A.; Theopold, K. H., A Chelating N-Heterocyclic Carbene Ligand in Organochromium. Chemistry. *Organometallics* **2006**, 25, 19, 4670–4679.

[52] Huffer, A.; Jeffery, B.; Waller, B. J.; Danopoulos, A. A., Synthesis of bis N-heterocyclic carbenes, derivatives and metal complexes. *C. R. Chimie* **2013**, 16, 557–565.

[54] Horrer, G.; Krummenacher, I.; Mann, S.; Braunschweig, H.; Radius, U., N-Heterocyclic carbene and cyclic (alkyl)(amino)carbene complexes of vanadium(III) and vanadium(V). *Dalton Trans.* **2022**, 51, 11054.

[58] Dresch, L. C.; Araujo, B. B. D.; Casagrande Jr., O. D. L.; Stieler, R., A novel class of nickel(II) complexes containing selenium-based bidentate ligands applied in ethylene oligomerization. *RSC Adv.* **2016**, 6, 104338.

[59] Thapa, R.; Kilyanek, S. M., Synthesis and structural characterization of nickel(II) complexes of 20-membered macrocyclic rings bearing chelating bis(N-heterocyclic carbene) ligands. *J. Organomet. Chem.* **2019**, 901, 120937.

^{1}H and ^{13}C NMR Spectra

Figure S4. ^{1}H and ^{13}C NMR of bis(imidazolium) salt 2a

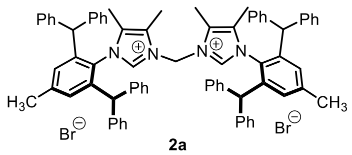

2a

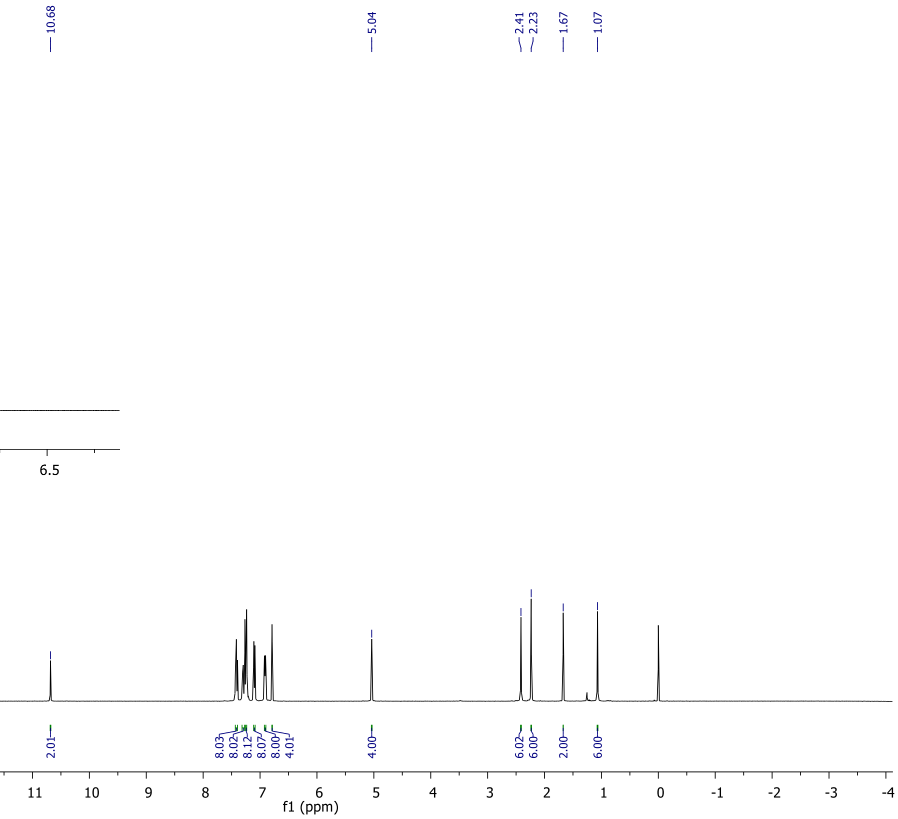

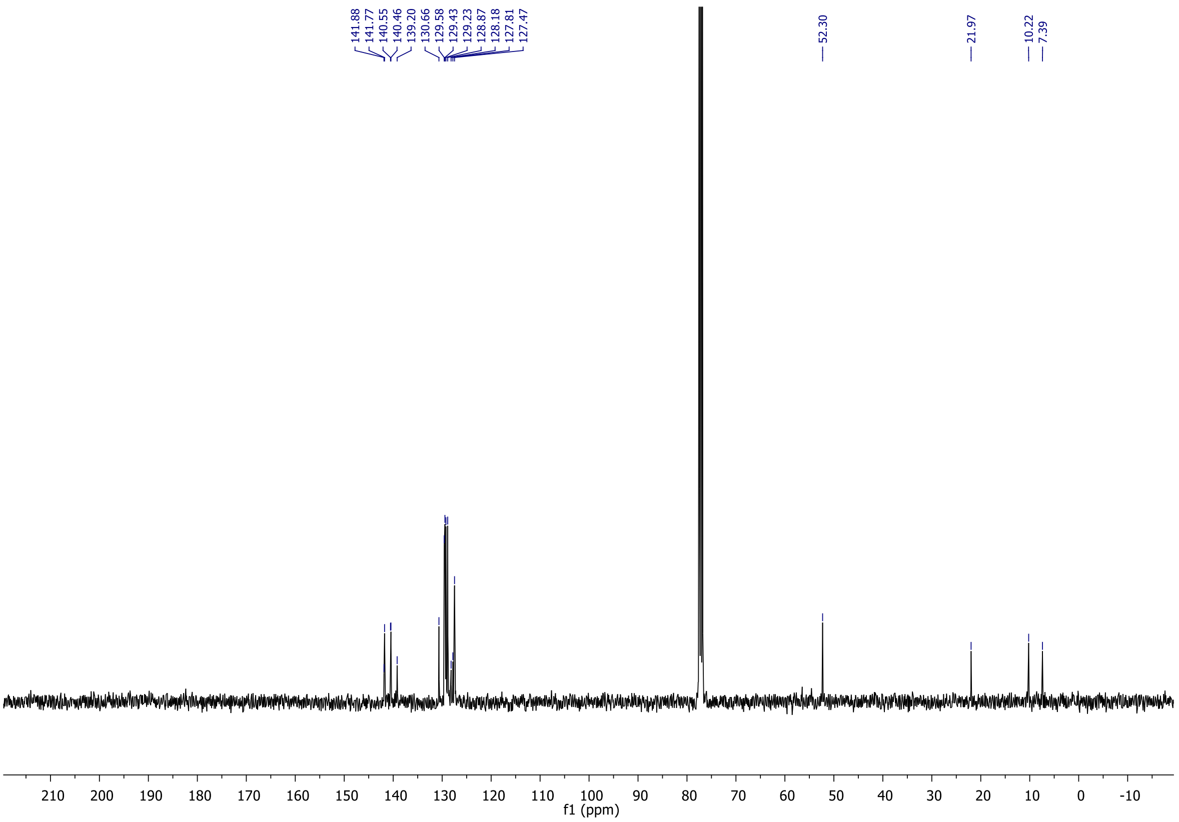

Figure S5. ^{1}H and ^{13}C NMR of polynorbornene obtained using vanadium(III) catalyst 3a

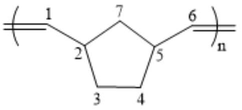

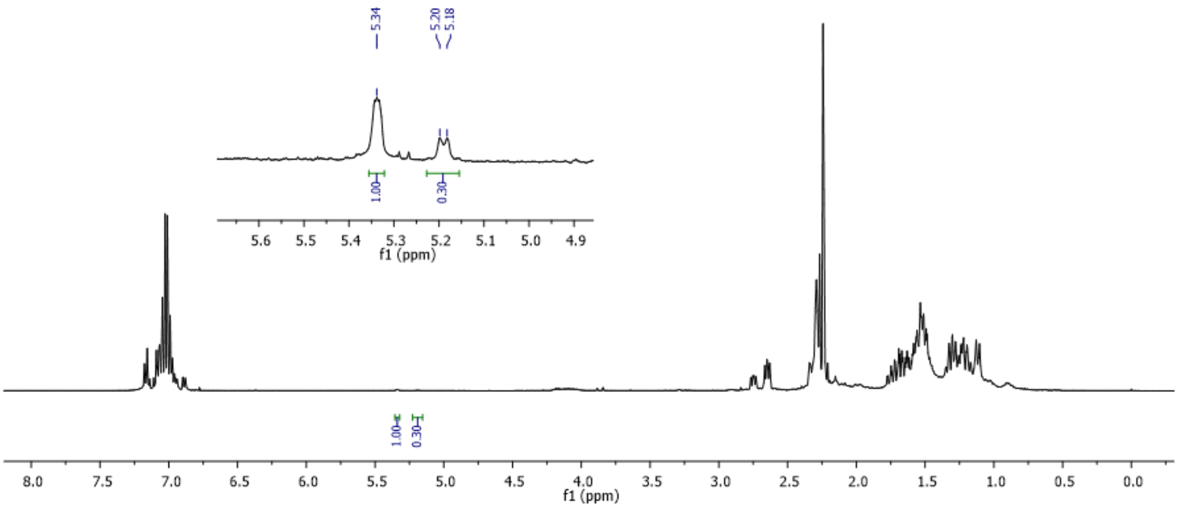

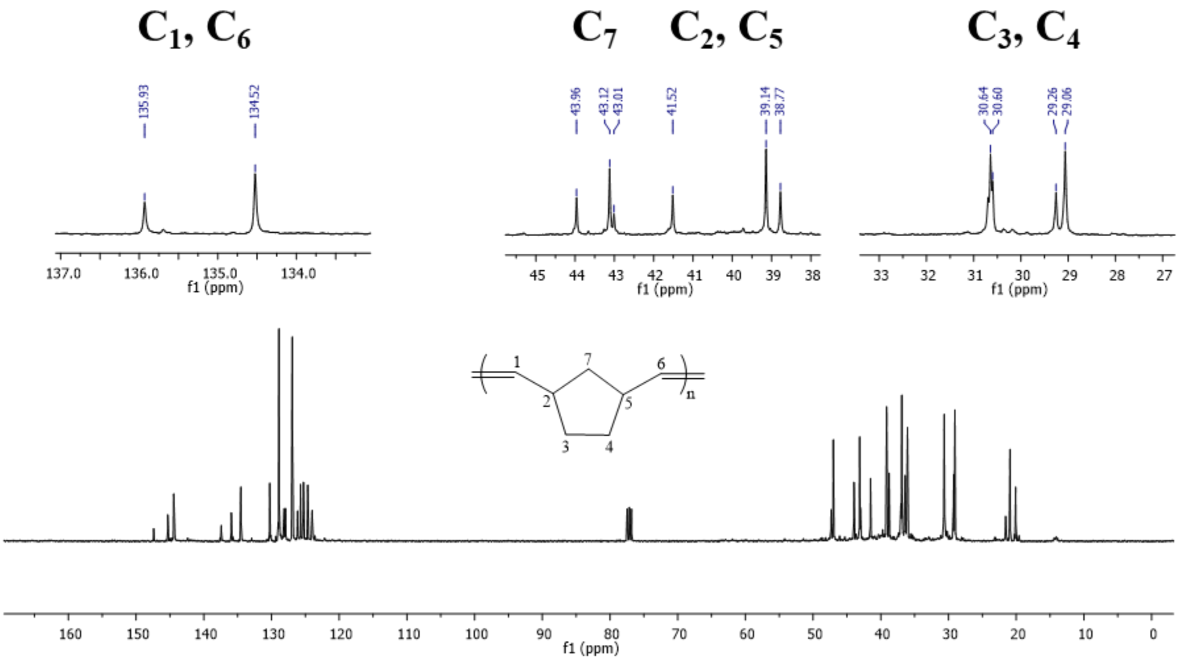

Figure S6. ^{1}H and ^{13}C NMR of polynorbornene obtained using vanadium(III) catalyst 3b

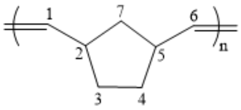

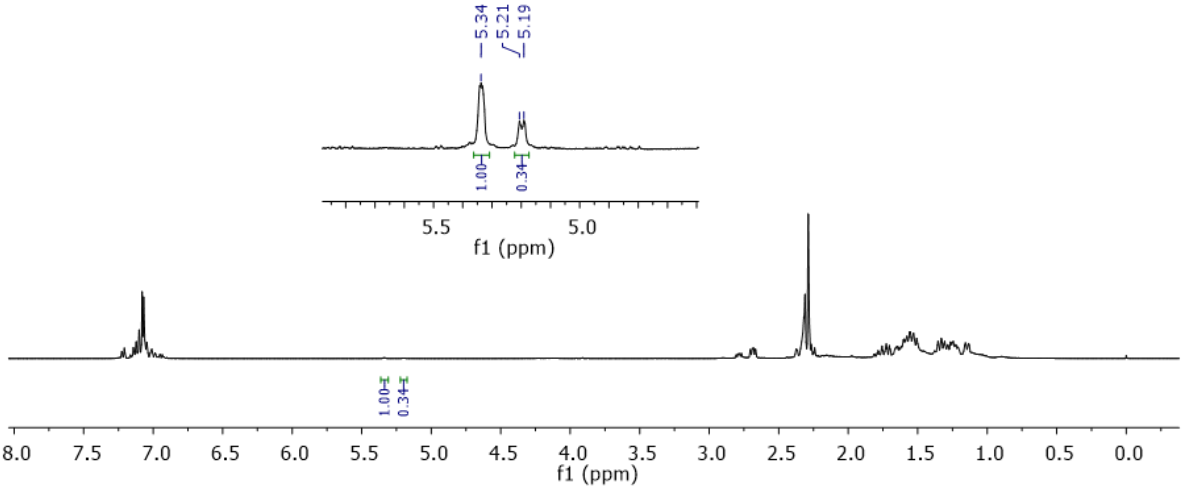

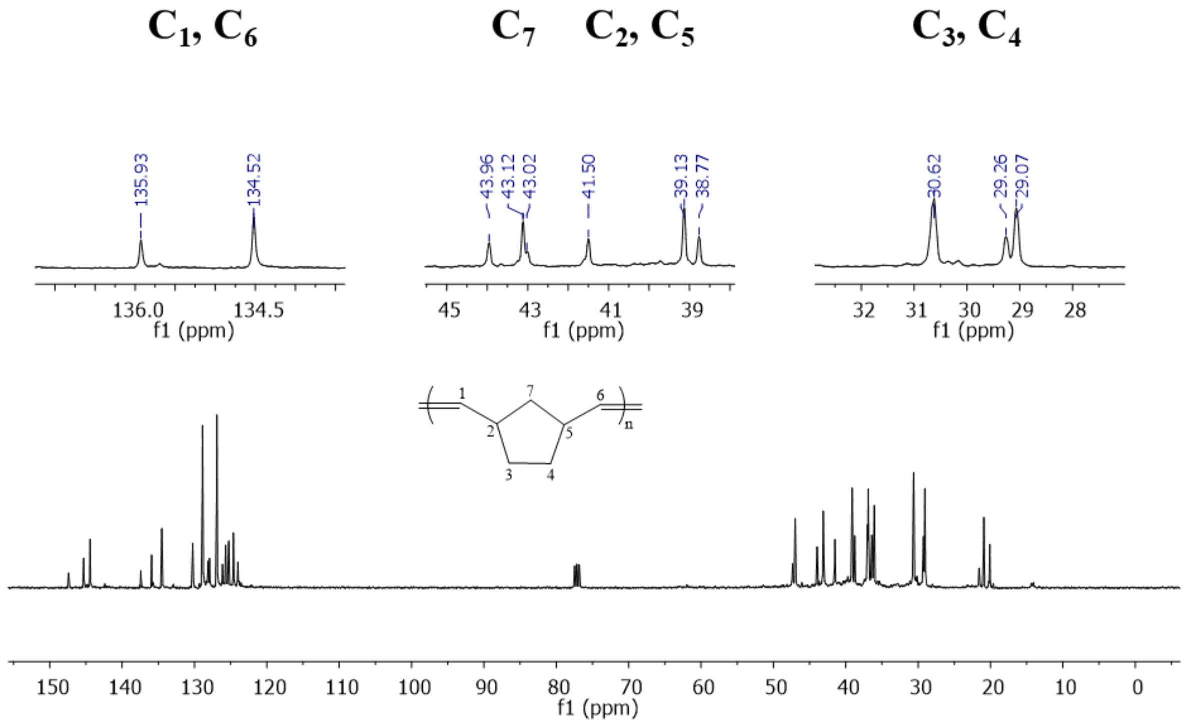

Figure S7. ^{1}H and ^{13}C NMR of polynorbornene obtained using nickel(II) catalyst 4a

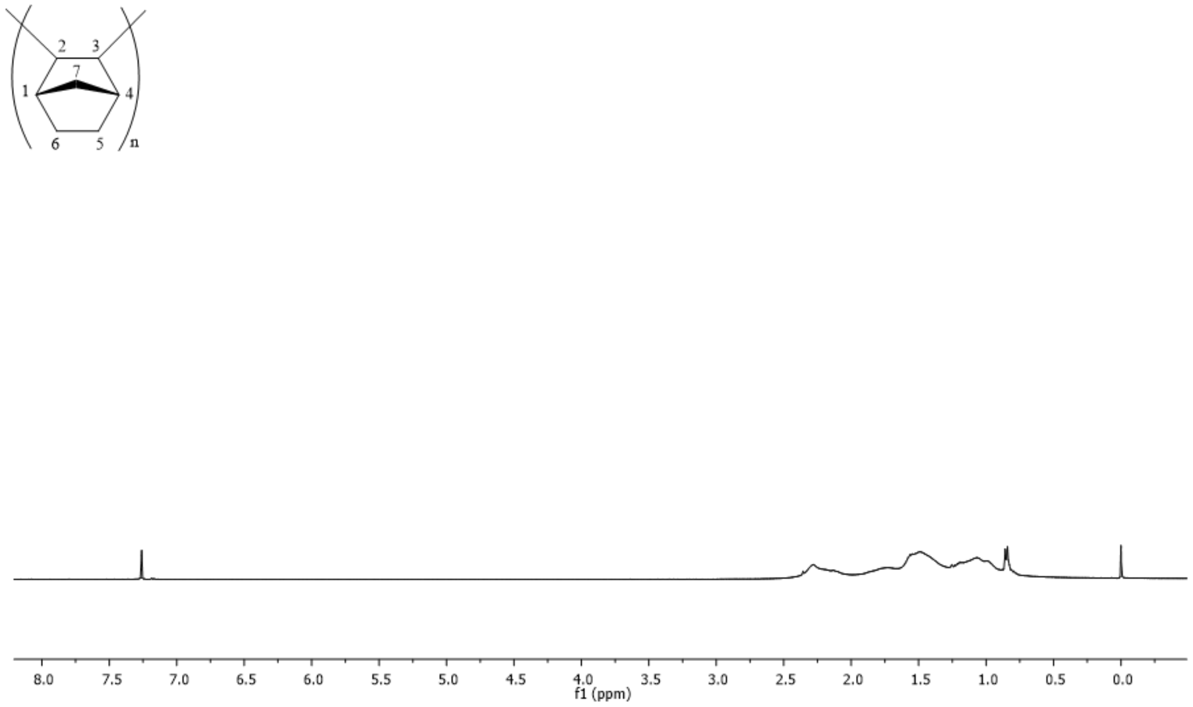

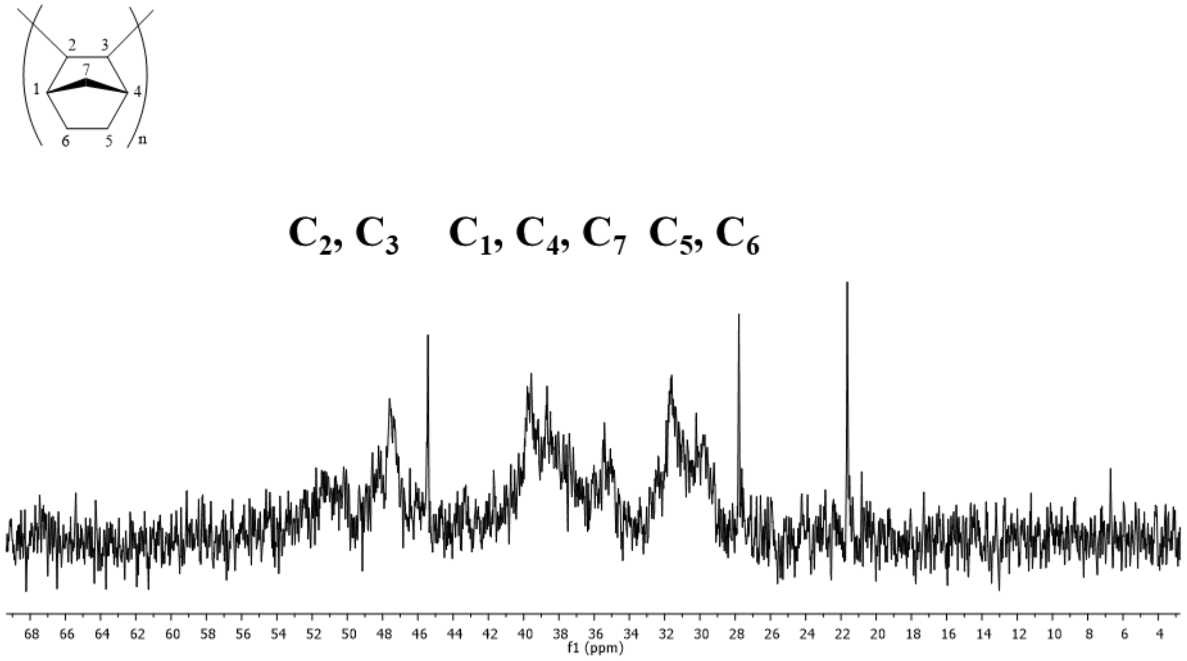

Figure S8. ^{1}H and ^{13}C NMR of polynorbornene obtained using nickel(II) catalyst 4b

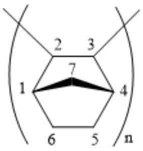

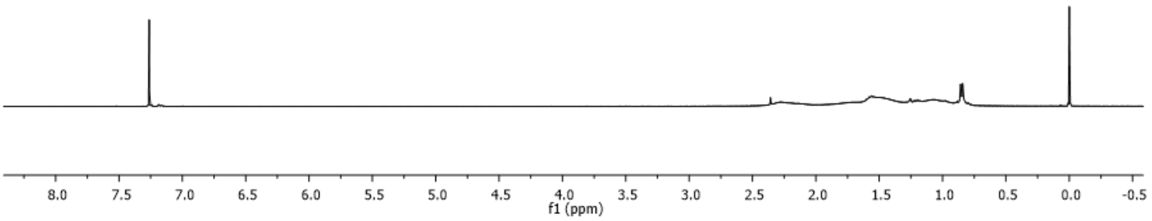

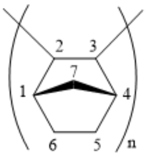

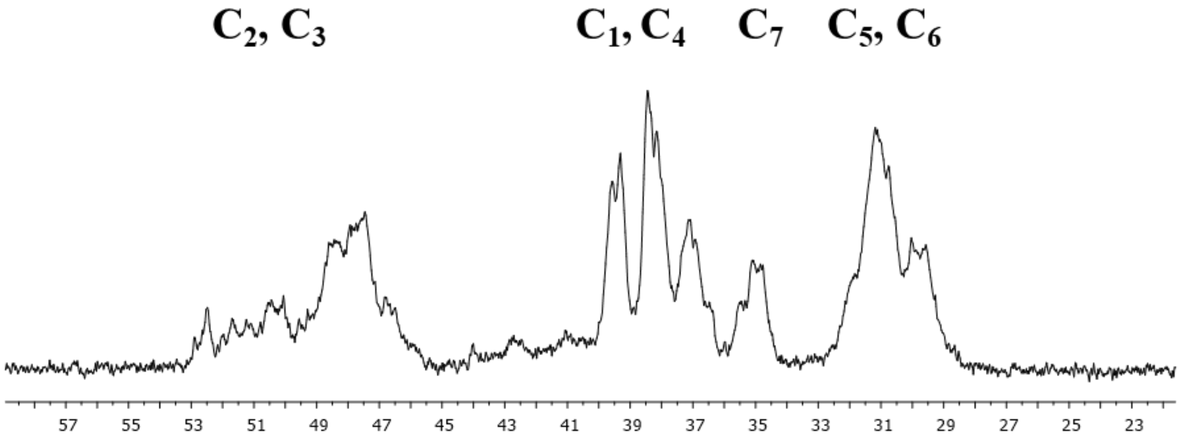

MS spectra

Figure S9. MS spectrum of pre-ligand 2a

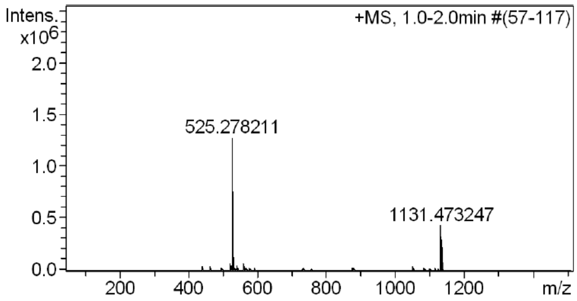

Figure S10. MS spectrum of vanadium(III) catalyst 3a

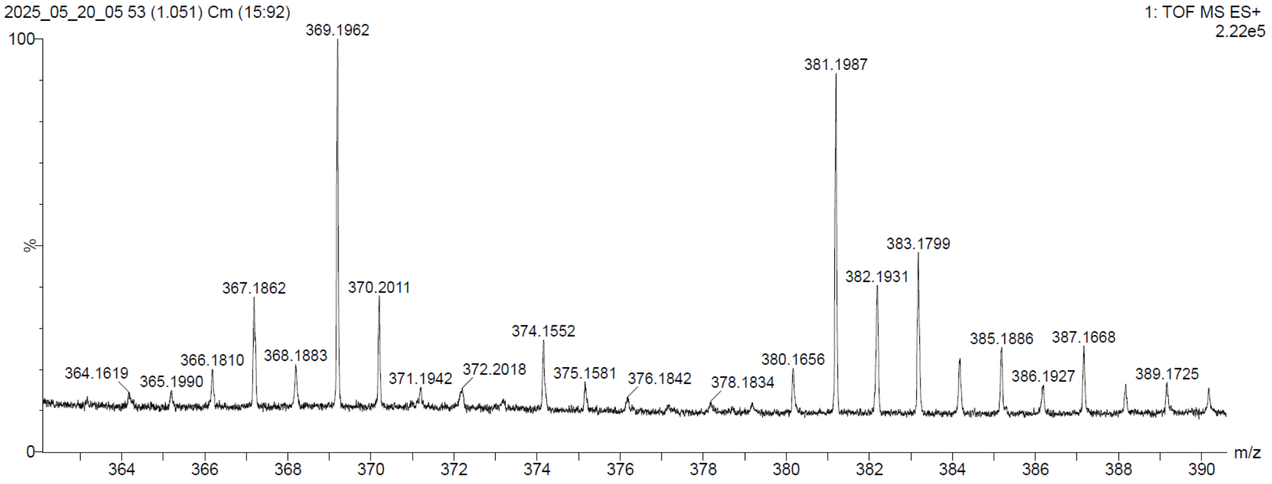

Figure S11. MS spectrum of vanadium(III) catalyst 3b

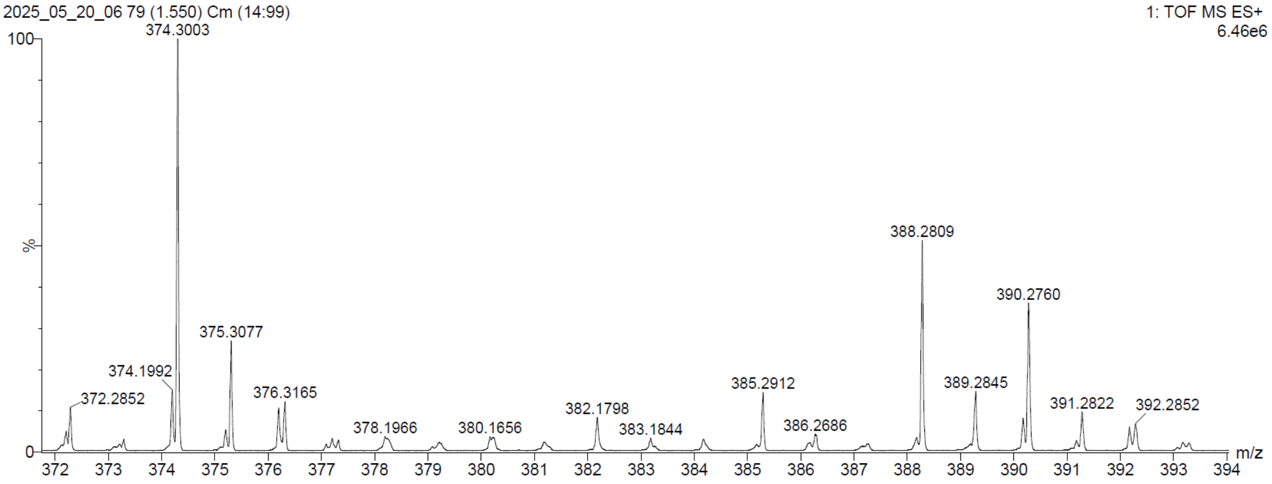

Figure S12. MS spectrum of nickel(II) catalyst 4a

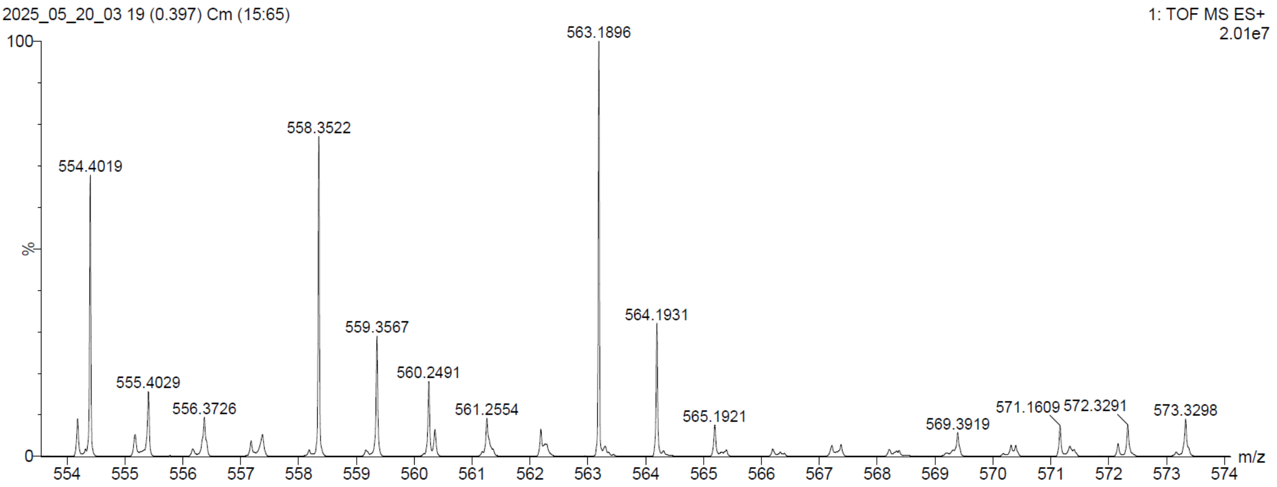

Figure S13. MS spectrum of nickel(II) catalyst 4b

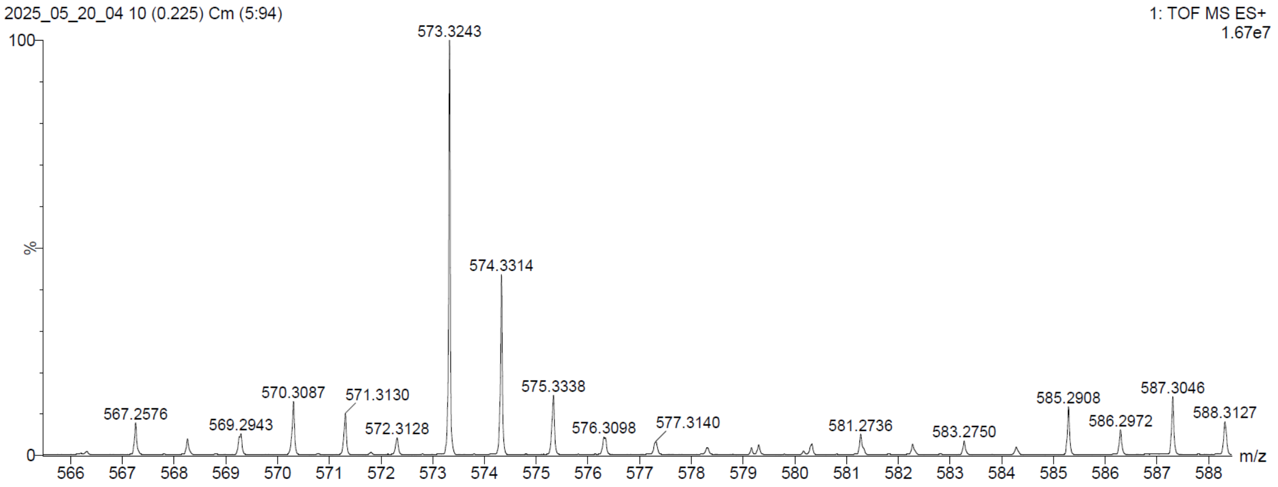

FTIR spectra

Figure S14. FTIR (ATR) of polynorbornene obtained using vanadium(III) catalyst 3b

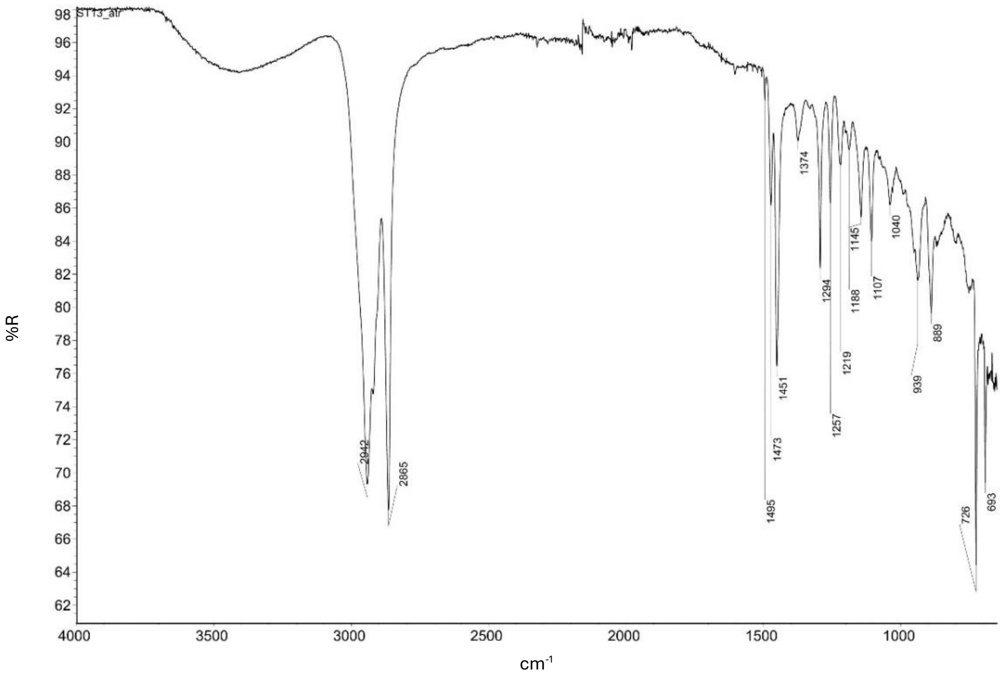

Figure S15. FTIR (ATR) of polynorbornene obtained using nickel(II) catalyst 4b

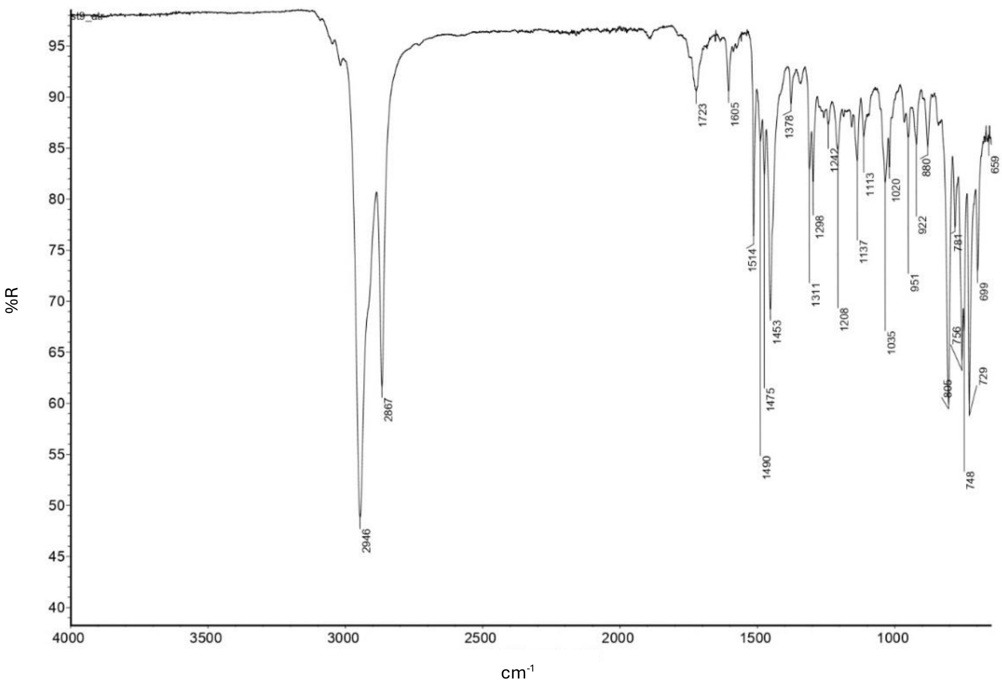

DSC and TGA analysis

Figure S16. Tm of PNB obtained using catalyst 3b

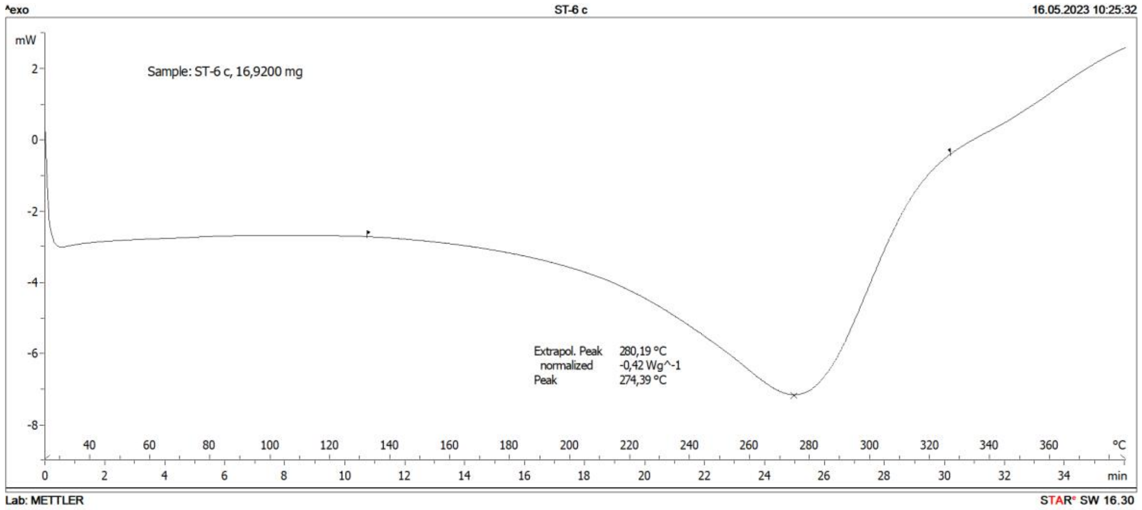

Figure S17. Tg of PNB obtained using catalyst 4a

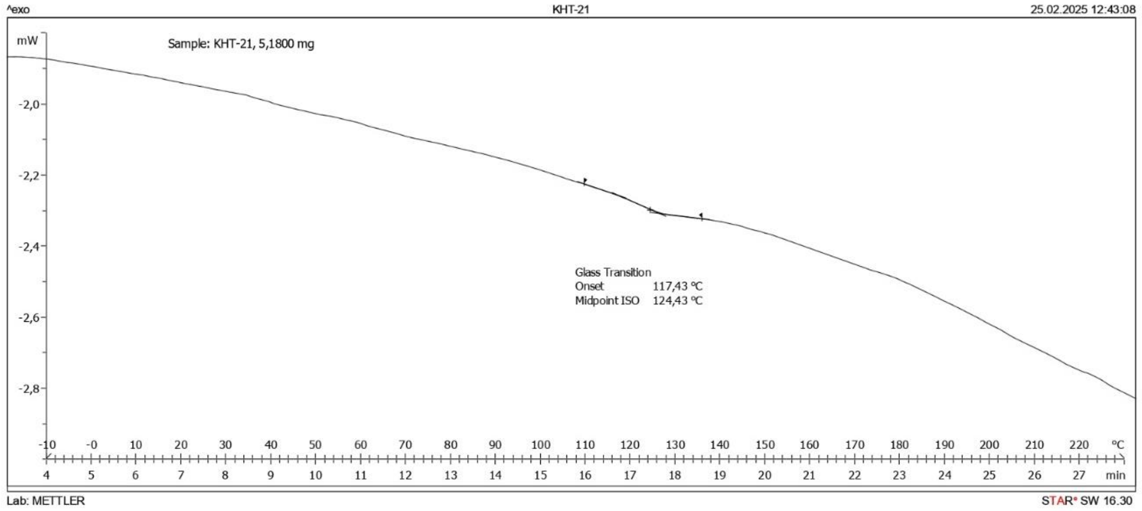

Figure S18. Tg of PNB obtained using catalyst 4b

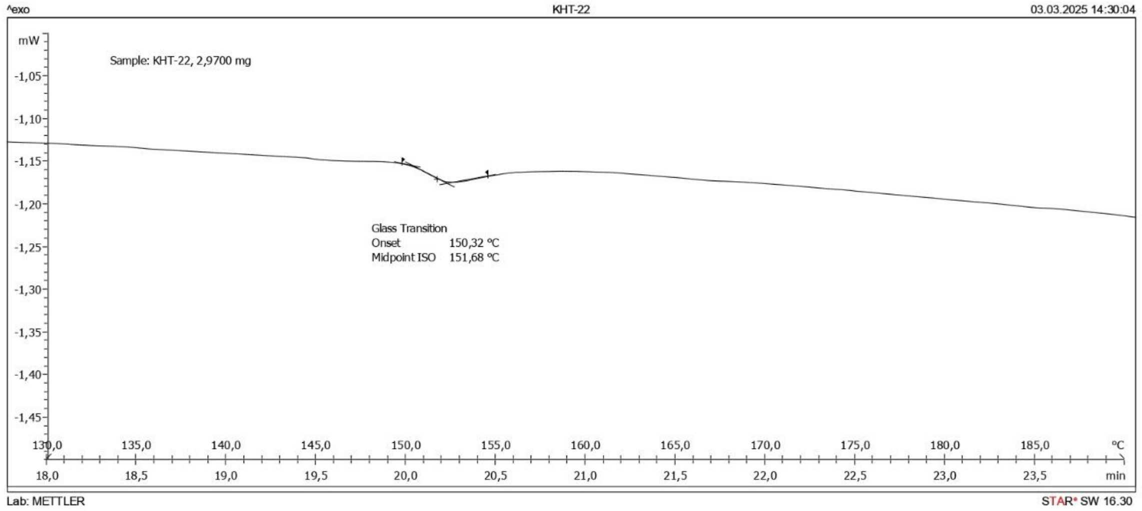

Figure S19. TGA analysis of PNB obtained using catalyst 4a

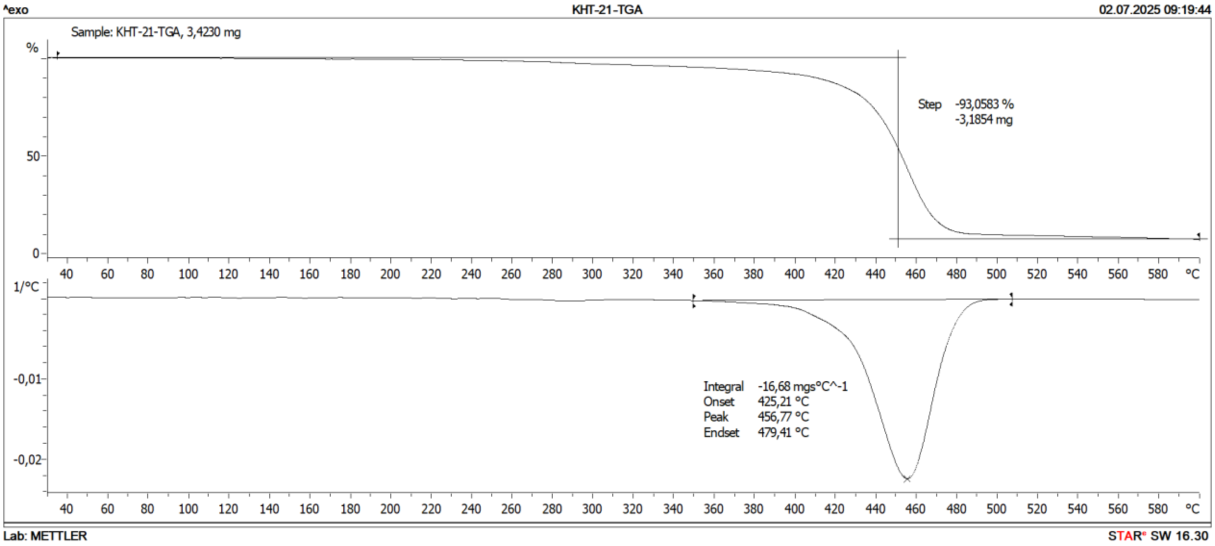

Figure S20. TGA analysis of PNB obtained using catalyst 4b

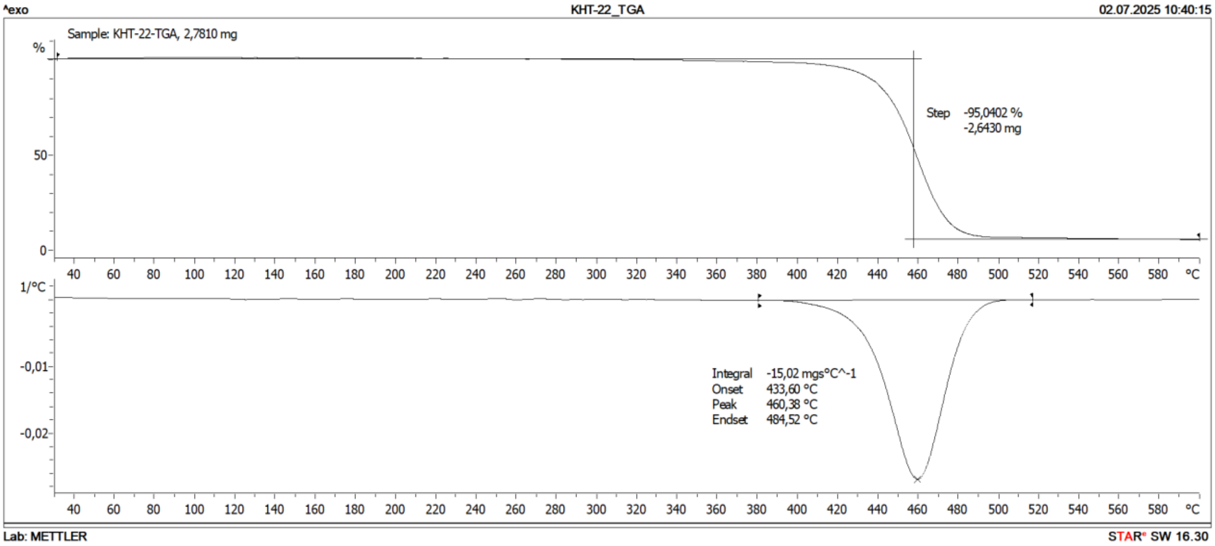

GPC Analysis

Figure S21. GPC Report of PNB obtained using catalyst 3a

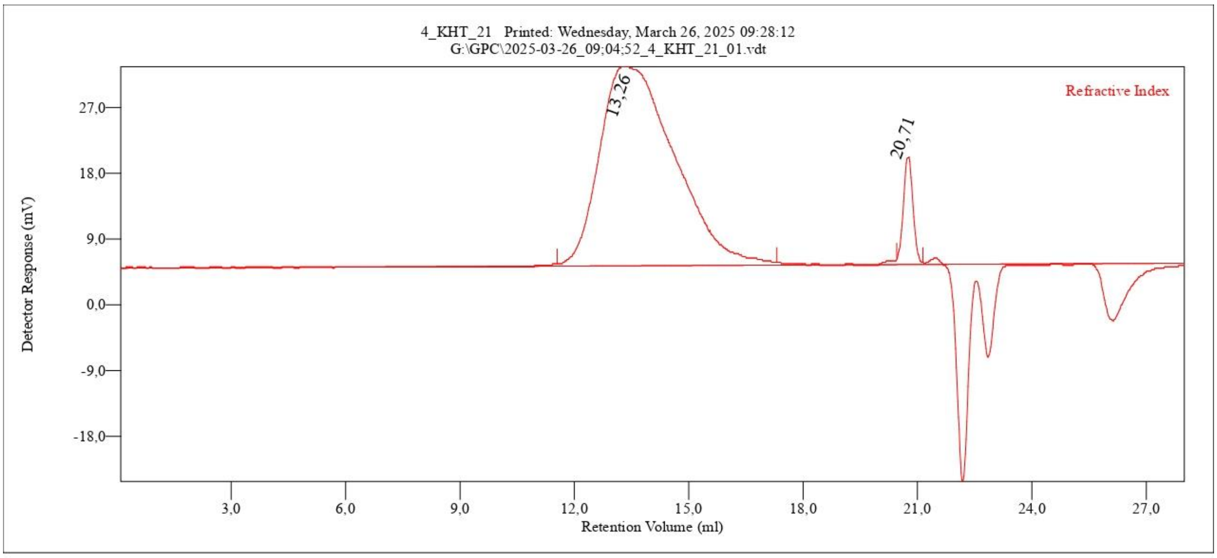

Conventional Calibration - Homopolymers : Results

Peak RV - (ml)	13,258	20,710
Mn - (Daltons)	29 870	0
Mw - (Daltons)	73 141	0
Mz - (Daltons)	125 361	0
Mp - (Daltons)	87 544	0
Mw / Mn	2,449	0,000
Percent Above Mw: 0	0,000	0,000
Percent Below Mw: 0	0,000	0,000
Mw 10.0% Low	8 822	0
Mw 10.0% High	208 957	0
RI Area - (mvml)	62,29	4,34
UV Area - (mvml)	0,00	0,00

Annotation	
Method File	4_CHCl3_20250324-0002.vcm
Limits File	52_4_KHT_21_01-4_CHCl3_20250324-0002-0000.lim
Date Acquired	Mar 26, 2025 - 09:04:52
Solvent	CHCl3
Acquisition Operator	admin : Administrator
Calculation Operator	admin : Administrator
Column Set	2xPlGelMixedC
System	CHCl3RIUV
Flow Rate - (ml/min)	1,000
Inj Volume - (ul)	100,0
Volume Increment - (ml)	0,01800
Detector Temp. - (deg C)	35,0
Column Temp. - (deg C)	35,0
OmniSEC Build Number	467

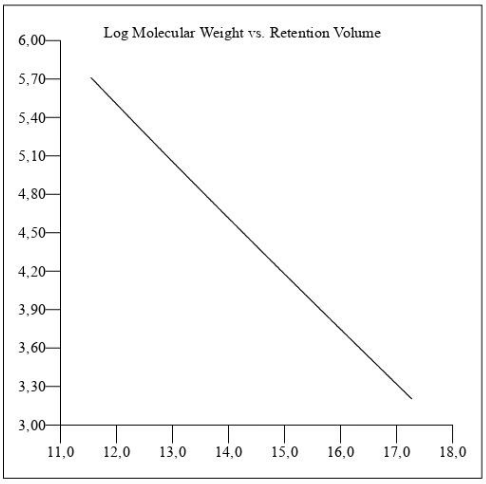

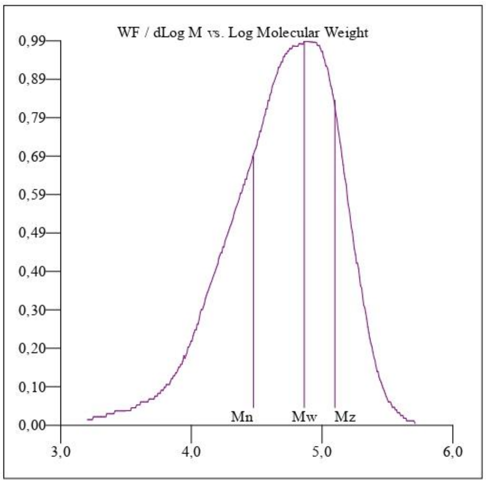

Figure S22. GPC Report of PNB obtained using catalyst 3b

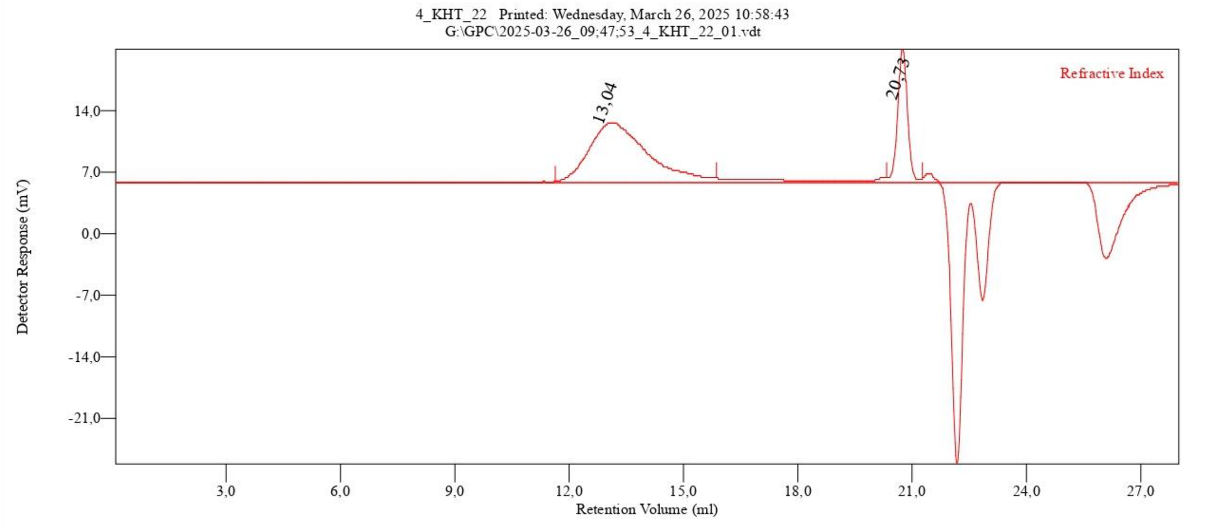

Conventional Calibration - Homopolymers : Results

Peak RV - (ml)	13,042	20,728
Mn - (Daltons)	49 958	0
Mw - (Daltons)	97 233	0
Mz - (Daltons)	148 111	0
Mp - (Daltons)	109 768	0
Mw / Mn	1,946	0,000
Percent Above Mw: 0	0,000	0,000
Percent Below Mw: 0	0,089	0,000
Mw 10.0% Low	14 487	0
Mw 10.0% High	250 341	0
RI Area - (mvml)	12,18	4,61
UV Area - (mvml)	0,00	0,00

Annotation	
Method File	4_CHCl3_20250324-0002.vcm
Limits File	53_4_KHT_22_01-4_CHCl3_20250324-0002-0000.lim
Date Acquired	Mar 26, 2025 - 09:47:53
Solvent	CHCl3
Acquisition Operator	admin : Administrator
Calculation Operator	admin : Administrator
Column Set	2xPlGelMixedC
System	CHCl3RIUV
Flow Rate - (ml/min)	1,000
Inj Volume - (ul)	100,0
Volume Increment - (ml)	0,01800
Detector Temp. - (deg C)	35,0
Column Temp. - (deg C)	35,0
OmniSEC Build Number	467

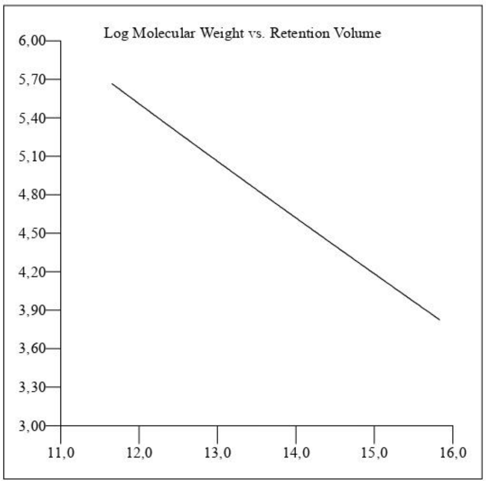

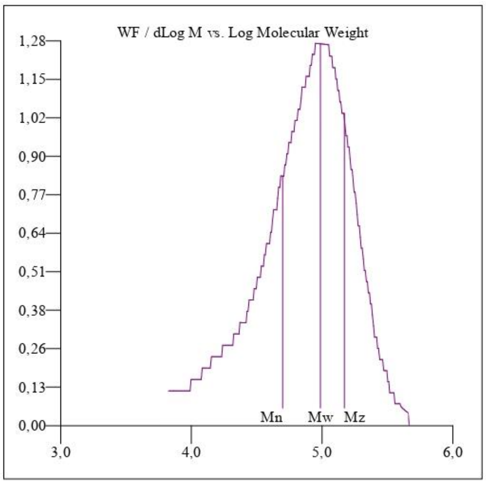

Figure S23. GPC Report of PNB obtained using catalyst 4a

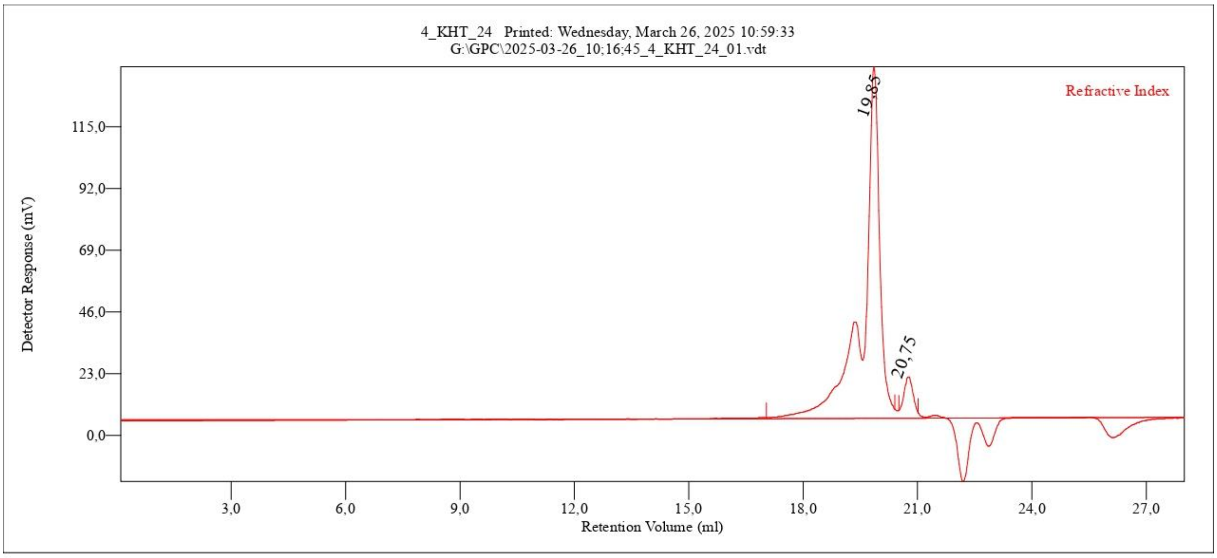

Conventional Calibration - Homopolymers : Results

Peak RV - (ml)	19,846	20,746
Mn - (Daltons)	157	0
Mw - (Daltons)	203	0
Mz - (Daltons)	343	0
Mp - (Daltons)	132	0
Mw / Mn	1,292	0,000
Percent Above Mw: 0	0,000	0,000
Percent Below Mw: 0	0,000	0,000
Mw 10.0% Low	101	0
Mw 10.0% High	585	0
RI Area - (mvml)	65,99	4,45
UV Area - (mvml)	0,00	0,00

Annotation	
Method File	4_CHCl3_20250324-0002.vcm
Limits File	
Date Acquired	Mar 26, 2025 - 10:16:45
Solvent	CHCl3
Acquisition Operator	admin : Administrator
Calculation Operator	admin : Administrator
Column Set	2xPlGelMixedC
System	CHCl3RIUV
Flow Rate - (ml/min)	1,000
Inj Volume - (ul)	100,0
Volume Increment - (ml)	0,01800
Detector Temp. - (deg C)	35,0
Column Temp. - (deg C)	35,0
OmniSEC Build Number	467

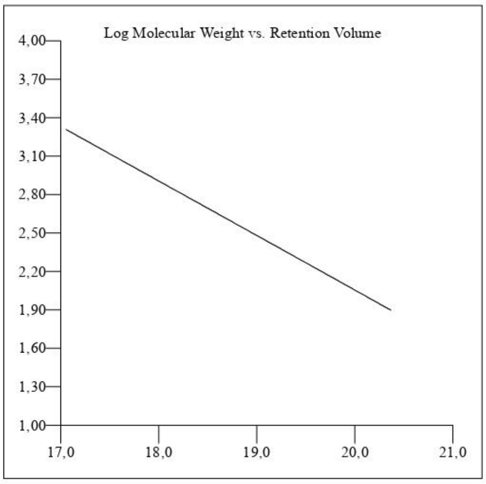

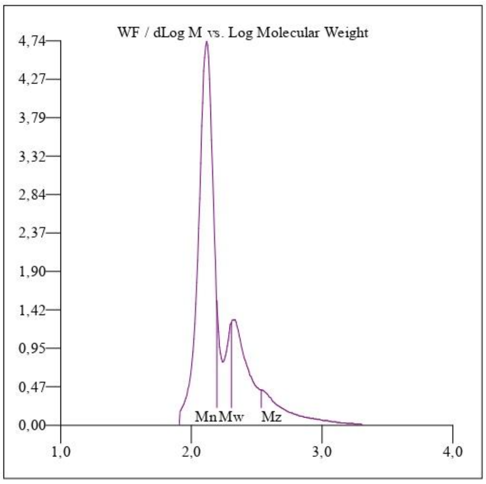

Figure S24. GPC Report of PNB obtained using catalyst 4b

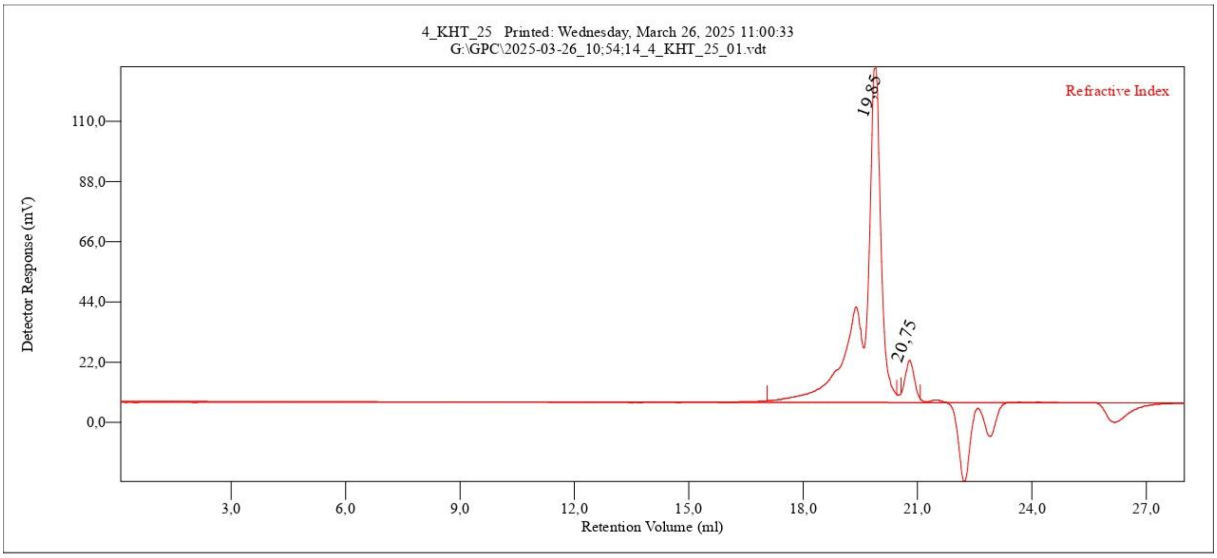

Conventional Calibration - Homopolymers : Results

Peak RV - (ml)	19,846	20,746
Mn - (Daltons)	157	0
Mw - (Daltons)	210	0
Mz - (Daltons)	383	0
Mp - (Daltons)	132	0
Mw / Mn	1,338	0,000
Percent Above Mw: 0	0,000	0,000
Percent Below Mw: 0	0,000	0,000
Mw 10.0% Low	100	0
Mw 10.0% High	643	0
RI Area - (mvml)	64,08	4,41
UV Area - (mvml)	0,00	0,00

Annotation	
Method File	4_CHCl3_20250324-0002.vcm
Limits File	
Date Acquired	Mar 26, 2025 - 10:54:14
Solvent	CHCl3
Acquisition Operator	admin : Administrator
Calculation Operator	admin : Administrator
Column Set	2xPlGelMixedC
System	CHCl3RIUV
Flow Rate - (ml/min)	1,000
Inj Volume - (ul)	100,0
Volume Increment - (ml)	0,01800
Detector Temp. - (deg C)	35,0
Column Temp. - (deg C)	35,0
OmniSEC Build Number	467

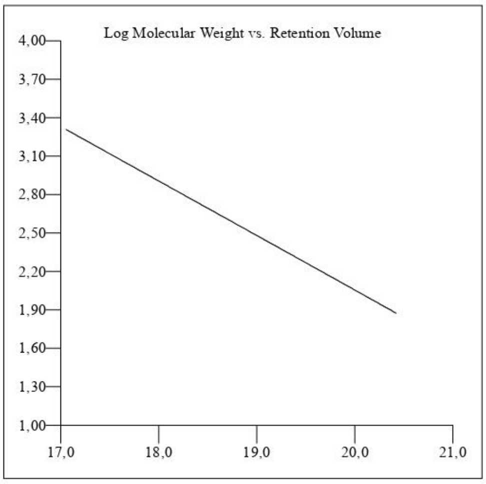

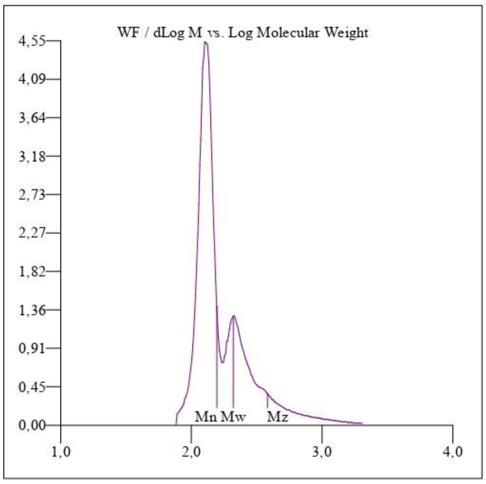

SEM imaging

Figure S25. SEM images of PNB obtained using catalyst 4a

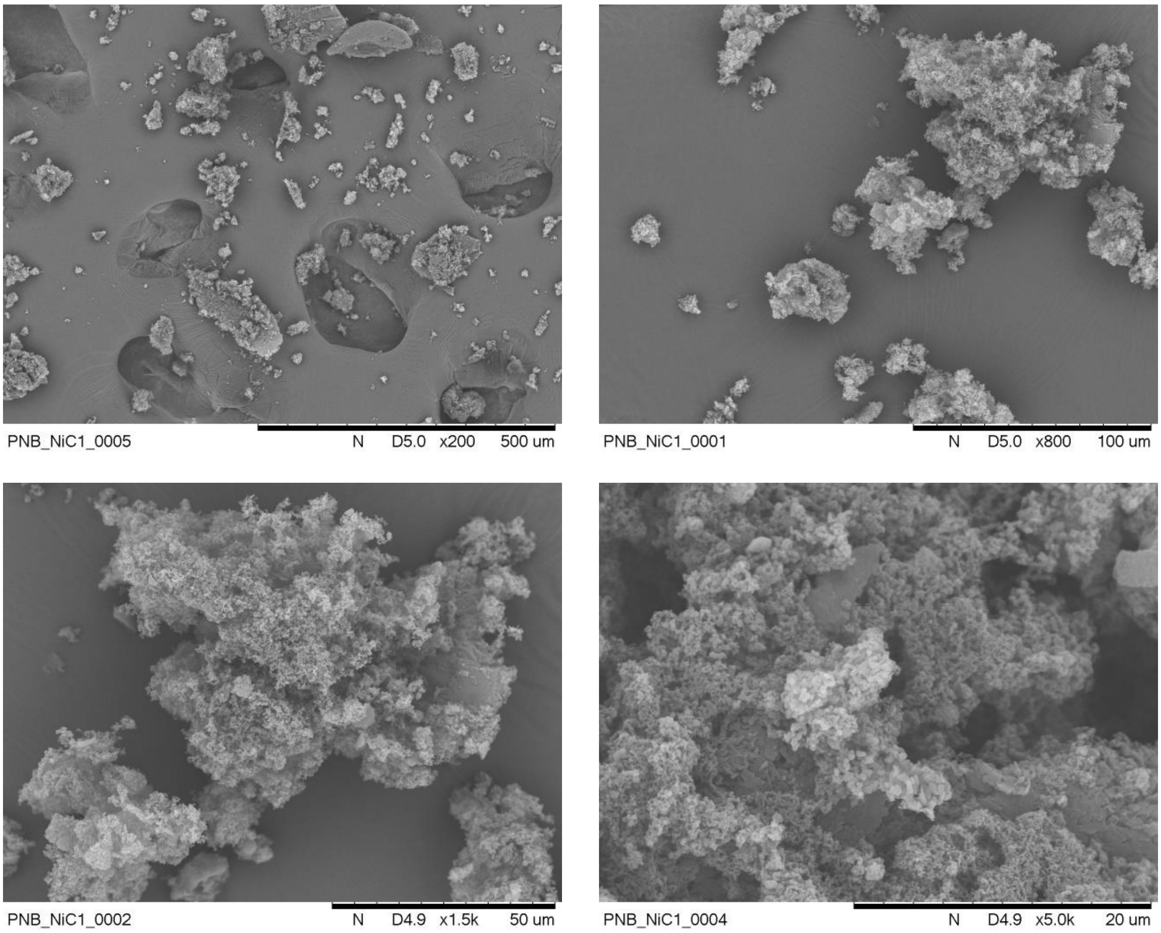

Figure S26. SEM images of PNB obtained using catalyst 4b

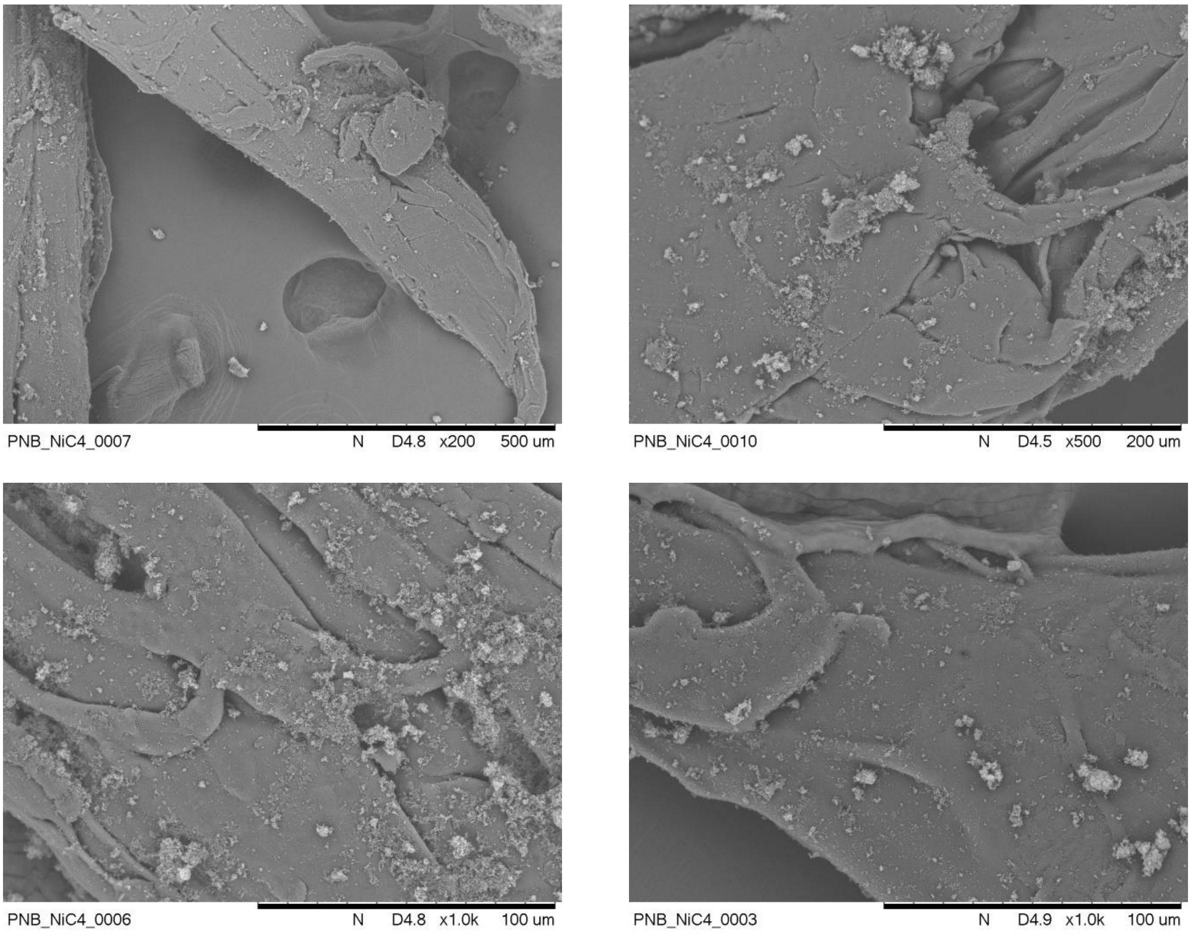

Supplement: Supplementary file 1 [file ijms-26-06691-s001.zip › ijms-3739206-supplementary.pdf]
